# Human Commensal *Prevotella histicola* Ameliorates Disease as Effectively as Interferon-Beta in the Experimental Autoimmune Encephalomyelitis

**DOI:** 10.3389/fimmu.2020.578648

**Published:** 2020-12-11

**Authors:** Shailesh K. Shahi, Samantha N. Jensen, Alexandra C. Murra, Na Tang, Hui Guo, Katherine N. Gibson-Corley, Jian Zhang, Nitin J. Karandikar, Joseph A. Murray, Ashutosh K. Mangalam

**Affiliations:** ^1^ Department of Pathology, University of Iowa, Iowa City, IA, United States; ^2^ Graduate Program in Immunology, University of Iowa, Iowa City, IA, United States; ^3^ Graduate Program in Molecular Medicine, University of Iowa, Iowa City, IA, United States; ^4^ Department of Immunology, Mayo Clinic, Rochester, MN, United States; ^5^ Division of Gastroenterology and Hepatology, Mayo Clinic, Rochester, MN, United States

**Keywords:** experimental autoimmune encephalomyelitis, human leukocyte antigen transgenic mice, multiple sclerosis, interferon beta, *Prevotella histicola*

## Abstract

Gut microbiota has emerged as an important environmental factor in the pathobiology of multiple sclerosis (MS), an inflammatory demyelinating disease of the central nervous system (CNS). Both genetic and environmental factors have been shown to play an important role in MS. Among genetic factors, the human leukocyte antigen (HLA) class II allele such as HLA-DR2, DR3, DR4, DQ6, and DQ8 show the association with the MS. We have previously used transgenic mice expressing MS susceptible HLA class II allele such as HLA-DR2, DR3, DQ6, and DQ8 to validate significance of HLA alleles in MS. Although environmental factors contribute to 2/3 of MS risk, less is known about them. Gut microbiota is emerging as an imporatnt environmental factor in MS pathogenesis. We and others have shown that MS patients have distinct gut microbiota compared to healthy control (HC) with a lower abundance of *Prevotella*. Additionally, the abundance of *Prevotella* increased in patients receiving disease-modifying therapies (DMTs) such as Copaxone and/or Interferon-beta (IFNβ). We have previously identified a specific strain of *Prevotella* (*Prevotella histicola*), which can suppress experimental autoimmune encephalomyelitis (EAE) disease in HLA-DR3.DQ8 transgenic mice. Since Interferon-β-1b [IFNβ (Betaseron)] is a major DMTs used in MS patients, we hypothesized that treatment with the combination of *P. histicola* and IFNβ would have an additive effect on the disease suppression. We observed that treatment with *P. histicola* suppressed disease as effectively as IFNβ. Surprisingly, the combination of *P. histicola* and IFNβ was not more effective than either treatment alone. *P. histicola* alone or in combination with IFNβ increased the frequency and number of CD4^+^FoxP3^+^ regulatory T cells in the gut-associated lymphoid tissue (GALT). Treatment with *P. histicola* alone, IFNβ alone, and in the combination decreased frequency of pro-inflammatory IFN-γ and IL17-producing CD4^+^ T cells in the CNS. Additionally, *P. histicola* alone or IFNβ alone or the combination treatments decreased CNS pathology, characterized by reduced microglia and astrocytic activation. In conclusion, our study indicates that the human gut commensal *P. histicola* can suppress disease as effectively as commonly used MS drug IFNβ and may provide an alternative treatment option for MS patients.

## Introduction

Multiple sclerosis (MS), an inflammatory and demyelinating disease of the central nervous system (CNS), is a multifactorial disease where the interaction between genetic and environmental factors play an important role in disease pathogenesis. Among various genetic factors (more than 50 genetic polymorphisms), human leukocyte antigen (HLA) class II haplotypes such as DR2/DQ6, DR3/DQ2, and DR4/DQ8 show the strongest association with MS (1, 2). Previously, we have characterized and validated HLA-DR3.DQ8 double transgenic mice as an animal model to study MS (3–6). HLA-DR3.DQ8 transgenic mice expressing the human class II genes HLA-DR3 (DRB1*0301) and DQ8 (DQB1*0302), lacking endogenous mouse class II genes (I-A and I-E) were used in this study.

Accumulating evidence suggests that environmental factors play an important role in a individual’s susceptibility to MS (7). However, specificity of an environmental factor contributing to MS susceptibility or resistance remains elusive. We and others have shown that the gut microbiota of MS patients are distinct from healthy controls (HC), suggesting the gut microbiota is an important environmental factor that contributes to MS pathogenesis (8–13). A number of MS microbiome studies have shown that genus *Prevotella* is either depleted or has a lower abundance among gut bacteria from MS patient, compared to HC (8–10, 14). Additionally, MS patients on disease-modifying therapies such as Copaxone or IFNβ showed a higher abundance of *Prevotella* compared to untreated MS patients (9, 15). We have previously identified a specific strain of *Prevotella*, *Prevotella histicola*, which can suppress proteolipid protein (PLP)_91-110_-induced EAE disease in the HLA-DR3.DQ8 transgenic mouse (5).

Interferon-β-1b [IFNβ (Betaseron)] is a major disease-modifying drug used in MS patients (16). The Food and Drug Administration (FDA)-approved an expanded repertoire of IFNβ use, namely intramuscular (IM) IFNβ-1a (Avonex, Biogen), subcutaneous (SC) IFNβ-1a (Rebif, EMD Serono), and PEGylated IFN (PEGIFN)- β-1a (Plegridy, Biogen) for the treatment of MS (17). Although IFNβ has been used as a first-line drug over the years in relapsing-remitting MS (RRMS), IFNβ alone is ineffective in 7–49% of patients with RRMS (18). Therefore, there is a need to develop additional therapeutic options that can either be used alone or in combination with IFNβ to improve the treatment for MS.

In the present study, we investigated whether a combination of *P. histicola* and IFNβ is more effective than either drug alone utilizing EAE in HLA-DR3.DQ8 transgenic mice. We found that *P. histicola* alone was as effective as IFN-β in suppressing PLP_91–110_-induced EAE in HLA-DR3.DQ8 transgenic mice. Additionally, we observe that naïve HLA-DR3.DQ8 transgenic mice treated with *P. histicola*, either alone or in combination with IFNβ, led to an increased frequency and number of CD4^+^Foxp3^+^ regulatory T cells (Treg) in the gut-associated lymphoid tissue (GALT). Treatment with *P. histicola* alone, IFNβ alone, and the combination of both in the EAE induction phase of disease also decreased frequency of pro-inflammatory IFN-γ and IL17-producing CD4^+^ T cells. Furthermore, we observed that *P. histicola* and*/*or IFNβ treatments suppressed microglia and astrocytes activation in the CNS. Altogether, our results suggest that *P. histicola* is as effective as IFNβ in suppressing EAE by boosting anti-inflammatory immune responses and inhibiting pro-inflammatory immune responses.

## Materials and Methods

### Mice

Human leukocyte antigen (HLA)-DR3.DQ8 double transgenic [DQ8 (DQA1*0103, DQB1*0302)-DR3 (DRB1*0301)] mice used in this study has been previously characterized and validated by our group (3–6, 19). The HLA-DR3.DQ8 mouse expresses the human class II genes HLA-DR3 (DRB1*0301) and DQ8 (DQB1*0302), and lack endogenous murine major histocompatibility complex (MHC) class II genes I-A, I-E (AE^−/−^). For the simplicity these mice will be referred as HLA-DR3.DQ8 transgenic mice throughout the text. Both male and female mice (8–12 weeks of age) were utilized in this study. Mice were bred and maintained in the University of Iowa animal facility in accordance with NIH and institutional guidelines. All experiments were approved by the Institutional Animal Care and Use Committee at the University of Iowa.

### Disease Induction in HLA-DR3.DQ8 Transgenic Mice and Scoring

HLA-DR3.DQ8 transgenic mice (8 to 12 weeks old) were immunized subcutaneously in both flanks with 25 µg of PLP_91–110_ emulsified in complete Freund’s adjuvant (CFA) containing *Mycobacterium tuberculosis* H37Ra (100 μg/mouse; Becton, Dickinson and Company, Sparks, MD, USA). Pertussis toxin (PTX) (Sigma Chemicals, St. Louis, MO, USA; 100 ng) was administered i.p. at days 0 and 2 post immunization. HLA-DR3.DQ8 transgenic mice were scored daily for clinical symptoms using the standard 0–5 scoring system described previously (5). Briefly, 0 for no disease; 1 for loss of tail tone; 2 for hind limb weakness; 3 for hind limb paralysis; 4 for hind limb paralysis and forelimb paralysis or weakness; and 5 for morbidity/death.

### Isolation, Characterization, and Identification of *Prevotella histicola*

Isolation, characterization, and identification (based on 16SrRNA-specific PCR) of *P. histicola* has been described previously (5). Briefly, *P. histicola* was grown at 37°C for 3 days in trypticase soy broth (TSB) (Hardy Diagnostics Santa Maria, USA) in an anaerobic jar with an AnaeroPack system (Mitsubishi Gas Chemical America) (5).

### Treatment of Mice With *Prevotella histicola* and IFNβ

We used two protocols for Interferon-beta (IFNβ) (Betaseron, Bayer HealthCare Pharmaceuticals) treatment: In the first protocol (prophylactic), IFNβ treatment was given every alternate day for 2 weeks to naïve HLA-DR3.DQ8 transgenic mice before the EAE induction and in second protocol IFNβ treatment was given during the disease induction phase (7 days post EAE induction).

In a prophylactic setting, HLA-DR3.DQ8 transgenic mice were divided into four groups (*P. histicola* alone, IFNβ alone, *P. histicola* and IFNβ, and media alone). IFNβ alone, *P. histicola* and IFNβ group of mice received 10,000 IU of IFNβ (20) in 100 µl of phosphate buffer saline (PBS) every other day for a total of seven doses. *P. histicola* alone and *P. histicola* plus IFNβ combination group of mice were orally gavaged with live *P. histicola* (10^8^ CFUs) every other day for a total of seven doses. Mice in the control group were orally gavaged with TSB media every other day for a total of seven doses.

In the second protocol, we treated mice in the induction phase (at disease onset) of the disease. Mice received 1^st^ dose of *P. histicola* treatment at day 7 post-immunization as the HLA-DR3.DQ8 transgenic mice develop the disease around day 7 (4, 6). Mice were divided into four groups (*P. histicola* alone, IFNβ alone, *P. histicola* plus IFNβ, and media alone) and treated on an alternate day with *P. histicola*, IFNβ, *P. histicola* plus IFNβ, or media as described above. All mice were evaluated for EAE scores till the duration of the experiment.

### Pathology

Brains and spinal cords from mice treated with *P. histicola* alone, IFNβ alone, a combination of both *P. histicola* plus IFNβ, or TSB media alone were fixed in 10% neutral buffered formalin, routinely processed and stained with Hematoxylin and Eosin (HE). Brains and spinal cords sections were analyzed for pathology, specially inflammation and demyleination, by a board-certified veterinary pathologist, specifically but not limited to the cortex, corpus callosum, hippocampus, brainstem, straitum, and cerebellum regions as described previously (4, 21).

### Immunohistochemistry (IHC)

Antigen retrieval was performed on freshly cut paraffin sections in a decloaking chamber for 5 min at 125°C in citrate buffer (pH 6.0). Endogenous peroxidase was blocked by incubation with 3% peroxide at room temperature for 8 min. For GFAP antibody staining (ab16997Abcam) the primary antibody was applied at 1:100 in Dako (Dako Agilent, Santa Clara, CA, USA) diluent for 1 h at room temperature after blocking with Dako Background Buster. IBA-1 immunostaining was performed with the antibody (019-10741, Wako Chemicals) diluted at 1:500 after blocking with both avadin/biotin block (Vector Laboratories) and 10% goat serum in Dako buffer. Bound antibody was detected using EnvisonTM + HRP, rabbit (Dako) for 30 min at room temperature followed by incubation with diaminobenzidene substrate (DAB) for 5 min at room temperature. Slides were counterstained with hematoxylin and evaluated by a board-certified veterinary pathologist (KGC).

### Western Blot Analysis

The brain and spinal cord’s tissues from mice were homogenized, and lysed in radioimmuno-precipitation assay (RIPA) buffer (25 mM Tris, pH 7.4, 150 mM NaCl, 5 mM EDTA, 1% Nonidet P-40, 1% sodium deoxycholate, 0.5% SDS, 100 µ M Na3VO4, 1 mM NaF, 1 mM PMSF, 10 µ g/ml aprotonin, 10 µg/ml leupeptin) (22). Protein concentrations in the tissue lysates were determined using NanoDrop Spectrophotometer (ThermoFisher; Waltham, MA, USA). Tissue lysates were normalized for protein concentration levels. Two hundred µg proteins were separated by SDSPAGE, and transferred to nitrocellulose membranes (Hybond C Super, cytiva, Marlborough, MA, USA). Membranes were blocked for 1 h at room temperature in TBST containing 5% milk, and then incubated overnight with anti-Iba1 (1:1,000; 016-20001, FUJIFILM Wako Pure Chemical Corporation; Richmond, VA, USA) or anti-GFAP (1:5,000; ab-254082, Abcam) at 4°C. After washing three times with TBS containing 0.05% Tween-20, membranes were incubated with HRP-conjugated mouse anti-rabbit IgG (1:30,000; #31464, ThermoFisher; Waltham, MA, USA), and visualized by Bio-Rad ChemiDocTM Touch Imaging System (Hercules, CA, USA).

### Flow Cytometry

Mononuclear infiltrating cells from the CNS (brain and spinal cord) were isolated using a percoll density gradient separation method as described previously (23). Mice in each treatment group were stained with antibodies to detect surface expression of CD4 (GK1.5) and CD25 (PC61) (BD Biosciences, Franklin Lakes, NJ, USA), whereas intracellular expression of FoxP3^+^ were stained using an anti-Mouse/Rat FoxP3 (FJK-16s) staining kit (eBiosciences, San Diego, CA, USA). Intracellular staining for IL17, IFN*γ*, GM-CSF, and IL10 were performed using the intracellular fixation permeabilization kit and anti-mouse IL17 (TC11-18H10.1), IFN*γ* (XMG1.2), GM-CSF (MP1-22E9), and IL10 (FES5-16E3) specific antibodies from eBioscience™. Cells were also stained with antibodies to detect surface expression of CD45 (30-F11) and CD4 (clone GK1.5) to gate on the leukocyte population. Gut-associated lymphoid cells were isolated and stained with antibodies as per the method described previously (24).

### Statistical Analysis

Differences in the frequency of regulatory T cells or cytokine-producing CD4 T cells among different treatment groups were assessed by Mann-Whitney U test. Average clinical EAE scores and cumulative EAE scores were compared using 2-way ANOVA with multiple comparisons of the means and non-parametric Mann-Whitney U test respectively. Statistical analyses were done with GraphPad Prism 8 (GraphPad Software, La Jolla, CA, USA). A value of p ≤ 0.05 was considered significant.

## Results

### *P. histicola* Suppresses EAE in Mice as Effectively as IFNβ

**Figure 1:**
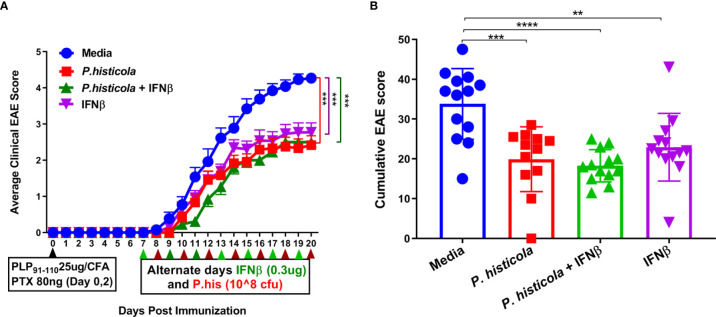
P*. histicola* suppresses PLP_91–110_-induced EAE in HLA-DR3.DQ8 transgenic mice as effectively as treatment with Interferon-Beta (IFNβ). **(A)** Mice were immunized with PLP_91–110_/CFA plus pertussis toxin on days 0 and 2 of the disease induction and 1 week later mice were treated with IFNβ, *P. histicola*, or a combination of both (with treatment administered on alternate days for a total of 14 doses, 7 doses of IFNβ and 7 doses of *P. histicola*) for 2 weeks. Clinical scores were assessed daily for the duration of the experiment. **(B)** Cumulative EAE scores of mice treated as in A. The data presented represent two of four experiments performed at different time points (n ≥ 12 mice per group). Two asterisks indicates p ≤ 0.01, three asterisks indicate p ≤ 0.001, and four asterisks indicate p ≤ 0.0001 when compared to the medium treated group. 2way ANOVA Dunnett’s multiple comparisons test were used to calculate *p*-value in average clinical EAE score **(A)** and Mann- Whitney unpaired U test was used to calculate *p-*value in cumulative EAE score **(B)**.

First, we examined whether the combination treatment of *P. histicola* and IFNβ can work in an additive manner to ameliorate disease in HLA-DR3.DQ8 transgenic mice. We started treating mice from day 7 postimmunization (disease induction phase) with *P. histicola* alone, IFNβ alone, and the combination of *P. histicola* and IFNβ. Treatment with *P. histicola* alone or IFNβ alone resulted in a lower average daily EAE score (**Figure 1A**) and a lower cumulative EAE score compared with mice who received media (**Figure 1B**). The combination treatment group had a similar average daily clinical score (**Figure 1A**) and average cumulative EAE score (**Figure 1B**) compared to the groups receiving *P. histicola* or IFNβ alone. Thus, our data indicate that *P. histicola* is effective at suppressing EAE when administered alone or in combination with IFNβ and is as effective as treatment with IFNβ alone.

### Treatment With *P. histicola* or IFNβ Reduces Inflammation in the CNS

**Figure 2:**
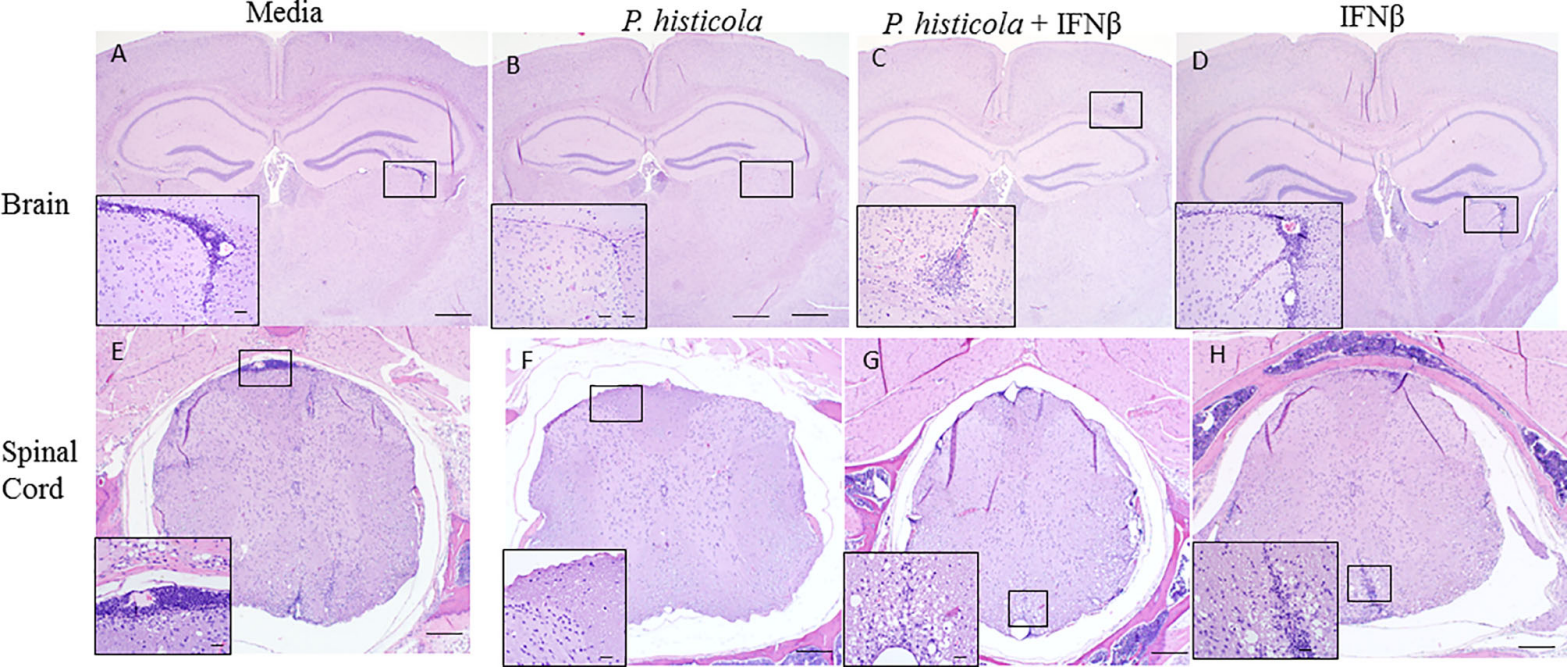
Treatment with *P. histicola* alone, IFNβ alone, or the combination of *P. histicola* and IFNβ resulted in decreased inflammation in the brain and spinal cord of mice induced with EAE. Representative HE stained images of the brain from mice treated with media **(A)**, *P. histicola* alone **(B)** or in combination with IFNβ **(C)**, and IFNβ alone **(D)**. Representative HE stained images of the lumbar spinal cord *in situ* from mice treated with media **(E)**, *P. histicola* alone **(F)** or in combination with IFNβ **(G)**, and IFNβ alone **(H)**. Insets in **(A–D)** identify areas of inflammatory cell infiltration and/or demyelination in brain (Bars = 500 um, inset bars = 50 um) and insets in E–H identify areas of inflammatory cell infiltration and/or demyelination in spinal cord when present (Bars = 200 um, inset bars = 20 um). The data presented represent one of two experiments performed at different time points (n ≥ 3 mice per group).

To determine whether disease suppression was accompanied with less severe CNS pathology, we analyzed brain and spinal cord tissues from all four groups by performing semi-quantitative analyses. HLA-DR3.DQ8 transgenic mice treated with *P. histicola* alone, or the combination of *P. histicola* and IFNβ had less inflammatory cell infiltrates in the brain and spinal cord compared to mice treated with media alone (**Figure 2**). Brain tissue sections from HLA-DR3.DQ8 transgenic mice treated with *P. histicola* alone, IFNβ alone, and the combination of both exhibited relatively less inflammation in the meningeal and stratum region compared with media group (**Figure 2**). Similarly, spinal cord tissue sections from mice that received *P. histicola* alone, or IFNβ alone and the combination of both, showed lower inflammation whereas spinal cord sections from mice treated with media showed severe inflammation (**Figure 2**). As mice received *P. histicola* orally, we sought to determine if *P. histicola* causes any histopathology (HE sections) of the gastrointestinal system. We found that neither of these treatment (*P. histicola* alone, IFNβ alone, and the combination of both) or the media control group caused any overt pathology in the stomach, small intestine, and large intestine (**Supplementary Figure 1**). In summary, we observed that treatment with *P. histicola* alone, IFNβ alone, or the combination of *P. histicola* plus IFNβ caused reduced CNS pathology in mice induced for EAE.

### Treatment With *P. histicola* or IFNβ Reduces Microglia and Astrocyte Activation

**Figure 3:**
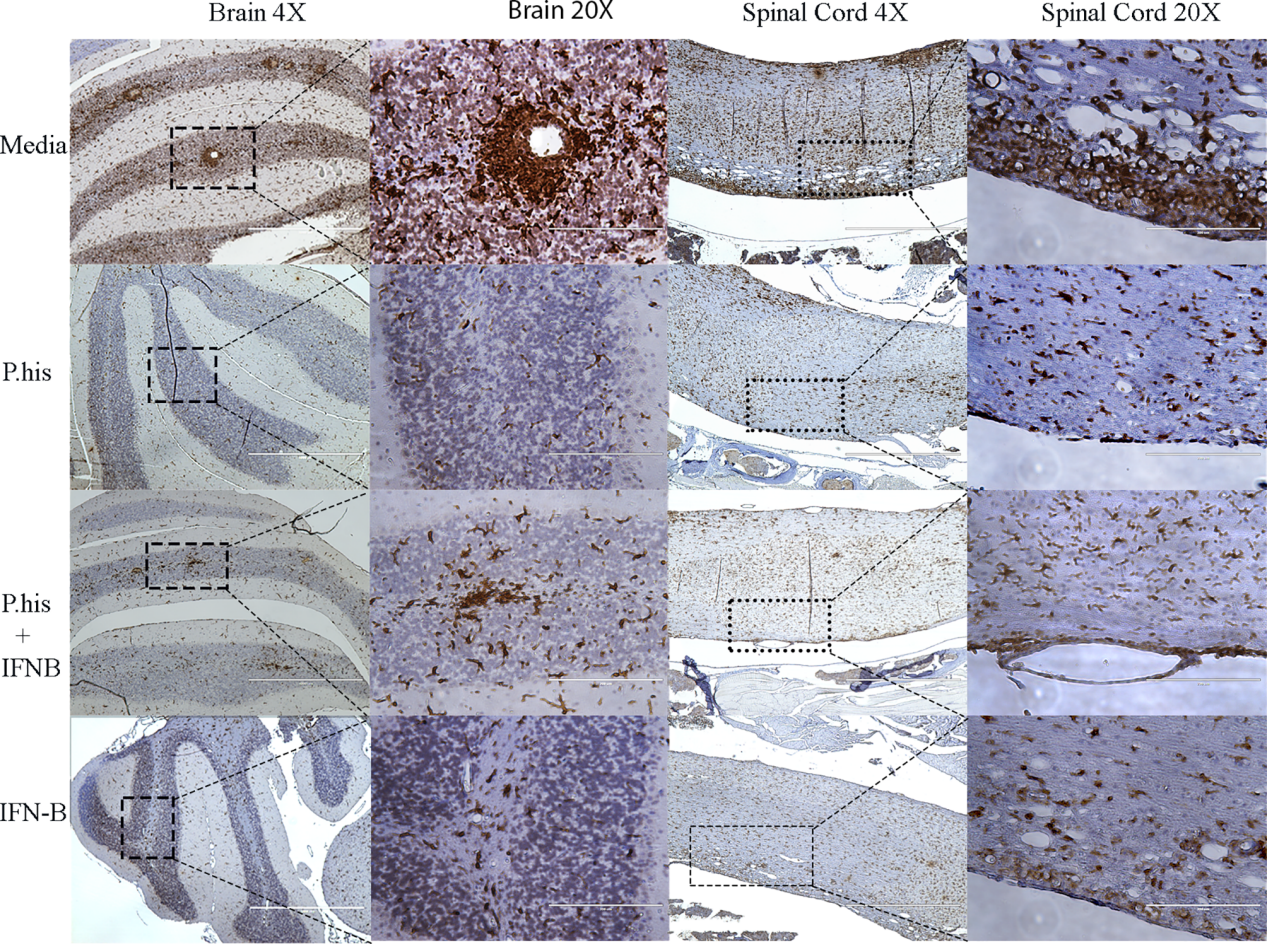
Treatment with *P. histicola* alone, IFNβ alone, or the combination of *P. histicola* and IFNβ resulted in decreased microglial activation in the brain and spinal cord of mice with EAE. Representative ionized calcium-binding adaptor protein-1 (Iba-1) staining of the brain and spinal cord sections from mice induced with EAE that received treatment with either *P. histicola* alone, IFNβ alone, the combination of both treatments, or media. The data presented represent one of two experiments performed at different time points (n ≥ 3 mice per group).

**Figure 4:**
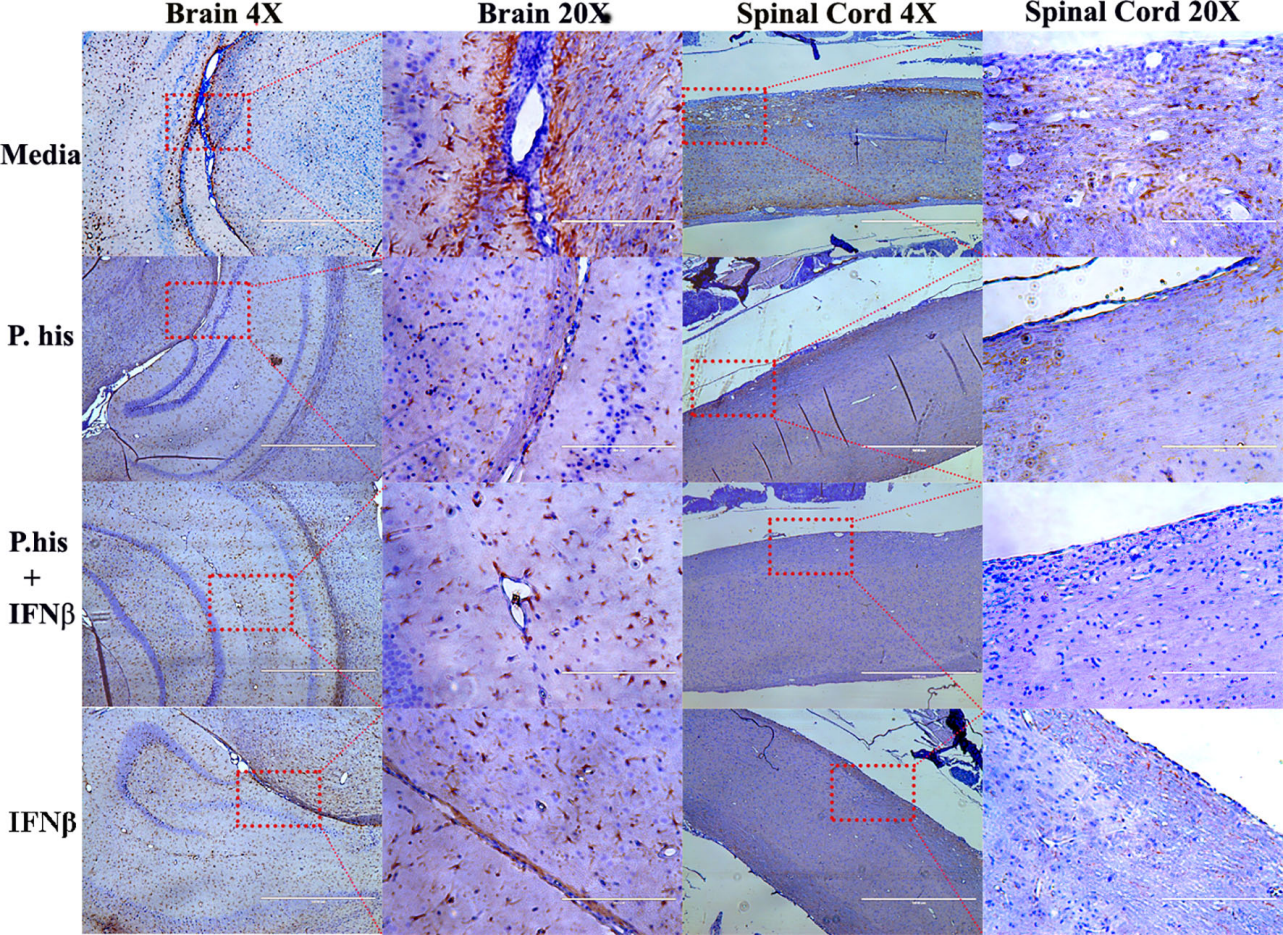
Treatment with *P. histicola* alone, IFNβ alone, or the combination of *P. histicola* and IFNβ reduced astrocytes activation in the brain and spinal cord of mice with EAE. Representative staining glial fibrillary acidic protein (GFAP) of the brain and spinal cord sections from mice induced with EAE that received treatment with either *P. histicola* alone, IFNβ alone, the combination with IFNβ, or media only. The data presented represent one of two experiments performed at different time points (n ≥ 3 mice per group).

The CNS resident microglia and astrocytes mediate myelin injury through enhanced phagocytosis and promoting autoreactive T-cell responses by functioning as antigen presenting cells (25, 26). Therefore, to determine whether *P. histicola* or IFNβ influence microglia and/or astrocyte activation, we stained CNS tissue with microglia specific anti-Iba-1 antibody and astrocyte specific GFAP antibody post-EAE induction. We found that the Iba-1 positive microglia cells were lower in the perivascular spaces of the brain cerebellar region and white matter of the spinal cord in treated group than those treated with media alone (**Figure 3**, **Supplementary Figure 2**). Additionally, a lower GFAP positive astrocytes were observed in cerebrum region of brain (**Figure 4**, **Supplementary Figure 3**) and white matter of the spinal cord (**Figure 4**) of the mice groups treated with *P. histicola* alone, IFNβ alone, or the combination of *P. histicola* plus IFNβ compared to the group treated with media alone. Besides IHC, expression of Iba-1 and GFAP at the protein level were confirmed in spinal cords by western blot using their specific antibodies. We observed that the Iba-1 and GFAP were lower at the protein levels in the spinal cord tissue from mice groups treated with *P. histicola* alone, IFNβ alone, or the combination of *P. histicola* plus IFNβ compared to the group treated with media alone (**Supplementary Figure 4**). Thus, treatment with *P. histicola* alone, IFNβ alone, or the combination of *P. histicola* plus IFNβ can reduce CNS pathology by reducing microglia and astrocytes activation in mice induced for EAE.

### *P. histicola* Alone and in Combination With IFNβ Induces CD4^+^FoxP3^+^ Regulatory T Cells in Gut-Associated Lymphoid Tissue of Mice

**Figure 5:**
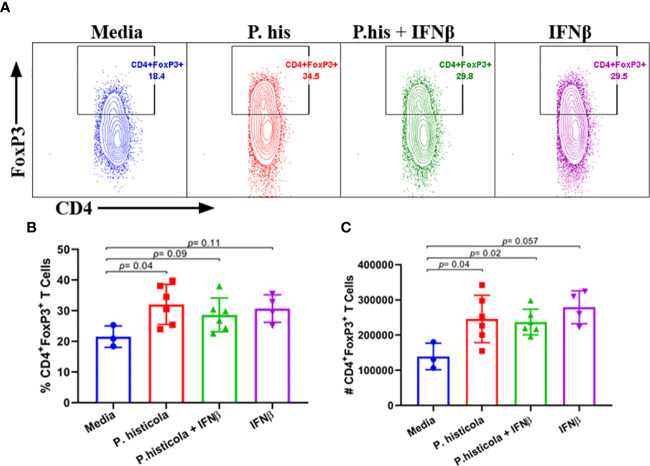
Pre-treatment with *P. histicola* alone or in combination with IFNβ increases CD4^+^FoxP3^+^ regulatory T cells in the gut-associated lymphoid tissue (GALT). **(A)** Naïve mice were treated with IFNβ (seven doses), *P. histicola* (seven doses), or a combination of both (with treatment administered on alternate days for a total of 14 doses). Gut-associated lymphoid cells were isolated from treated and the control group of mice and stained with CD45, CD4, and FoxP3 antibodies. Representative flow cytometric plots to demonstrate CD4^+^FoxP3^+^ regulatory T cells in GALT of mice treated with *P. histicola* alone, IFNβ alone, *P. histicola* and IFNβ, or media. **(B)** Frequency of CD4^+^FoxP3^+^ regulatory T cells from mice treated IFNβ, *P. histicola*, *P. histicola* plus IFNβ, and media. **(C)** Quantification of the number of CD4^+^FoxP3^+^ regulatory T cells in mice treated as in **(A)**. Error bars are presented as the standard error of the mean. The *p*-value determined by the Mann-Whitney unpaired U test for comparing each group to media. Criteria for setting positive gates for specific flourochrome and gating strategy for different cell populations had been provided in **Supplementary Figure 6**. The data presented represent one of three experiments performed at different time points (n ≥ 3 mice per group).

**Figure 6:**
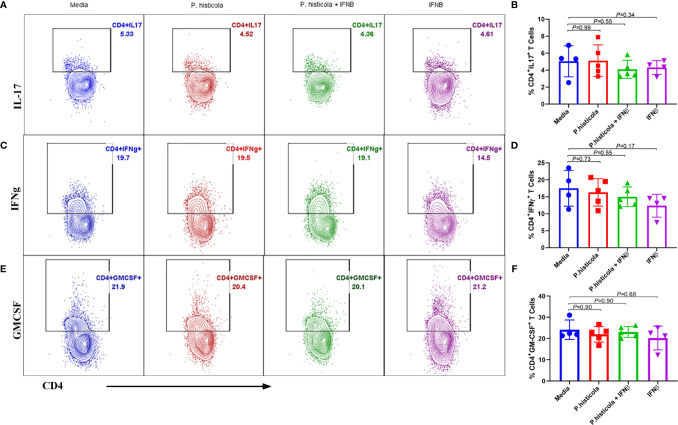
Treatment with *P. histicola* alone, IFNβ alone or *P. histicola* plus IFNβ do not modulate CD4^+^IL17^+^, CD4^+^IFNγ^+^, and CD4^+^GM-CSF^+^ cells frequency in the GALT of naïve mice. Mice were treated with IFNβ (seven doses), *P. histicola* (seven doses), or a combination of both (with treatment administered on alternate days for a total of 14 doses, 7 doses of IFNβ and 7 doses of *P. histicola*). Flow cytometric were done to study IL17^+^, IFNγ^+^, and GM-CSF^+^ expressing CD4 T cells that were isolated from gut-associated lymphoid tissue of mice treated as mentioned. Cells were previously gated on lymphocytes and singlets. **(A)** Representative flow cytometric plots to demonstrate CD4^+^IL17^+^ T cells in the GALT of mice treated with *P. histicola* alone, IFNβ alone, *P. histicola* and IFNβ, or media. **(B)** Frequency of CD4^+^IL17^+^ T cells from mice treated as in **(A)**. **(C)** Representative flow cytometric plots to demonstrate CD4^+^ IFNγ^+^ T cells in the GALT of mice treated as in **(A)**. **(D)** Frequency of CD4^+^ IFNγ^+^ T cells from mice treated as in **(A)**. **(E)** Representative flow cytometric plots to demonstrate CD4^+^GM-CSF^+^ T cells in the GALT of mice treated as in **(A)**. **(F)** Frequency of CD4^+^GM-CSF^+^ T cells from mice treated as in **(A)**. Gating strategy for different population had been provided in **Supplementary Figure 7**. Error bars presented as standard error of the mean. *P*-value determined by Mann-Whitney unpaired t-test for comparing each group to media.

We and others have shown that CD4^+^FoxP3^+^ Treg cells play a significant role in suppressing EAE disease (5, 6, 27). As *P. histicola* is a gut commensal, it may modulate the immune system through influencing the immune compartment of the intestinal tract. Therefore, we analyzed levels of CD4^+^FoxP3^+^ Treg cells and CD4^+^ IL10^+^ T cells in the GALT. Mice treated with *P. histicola* alone showed a higher frequency and number of CD4^+^FoxP3^+^ Treg cells compared to mice treated with media (*%* mean 21.53 *vs.* 32.05 ± 3.87, *p* = 0.037, number mean 139,241 *vs.* 245,810 ± 35,924, *p* = 0.04) (**Figures 5A–C**). Mice treated with a combination of *P. histicola* and IFNβ also showed the higher number but not the frequency of CD4^+^FoxP3^+^ Treg cells compared to media treated group (*%* mean 21.53 *vs.* 28.65 ± 3.87, *p* = 0.09, # mean 139,241 *vs.* 237,258 ± 35,924, *p* = 0.02) (**Figures 5A–C**). We did not observe any change in CD4^+^FoxP3^+^ Treg cells in the IFNβ alone treated mice group compared to media control group (*%* mean 21.53 *vs.* 30.73 ± 0, *p* = 0.11, # mean 139,241 *vs.* 279,087 ± 38,803, *p* = 0.057) (**Figures 5A–C**). Additionally, we found that the *P. histicola* alone or in combination with IFNβ treatment led to a higher number and frequency of CD4^+^ IL-10 T cells compared with media control group (**Supplementry Figure 5**). We did not observe any difference in IL-17, IFN-γ, and GM-CSF, producing CD4^+^ T cells in the GALT (**Figures 6A–F**). Thus, our data indicate that *P. histicola* alone or in combination with IFNβ, ameliorates disease and is associated with an induction of CD4^+^FoxP3^+^ regulatory T cells and CD4^+^ IL10^+^ T cells in the gut. However, IFN-γ alone had no effect on either CD4^+^FoxP3^+^ regulatory T cells or CD4^+^ IL-10 T cells in the gut.

### Treatment With *P. histicola* and/or IFNβ Reduces Antigen-Specific Th1 and Th17 Cytokines in the CNS of Mice Induced With EAE

**Figure 7:**
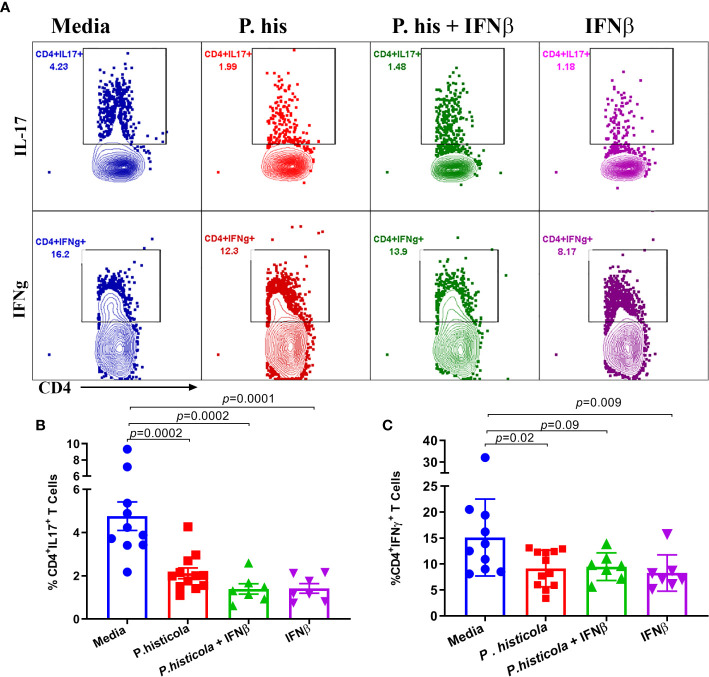
Treatment with *P. histicola* alone, IFNβ alone or *P. histicola* plus IFNβ modulate CD4^+^IL17^+^, and CD4^+^IFNγ^+^, T cells frequency in the CNS of mice. Mice were immunized with PLP_91–110_/CFA plus pertussis toxin on days 0 and 2 of the disease induction and 1 week later mice were treated with IFNβ (seven doses), *P. histicola* (seven doses), or a combination of both (with treatment administered on alternate days for a total of 14 doses, 7 doses of IFNβ and 7 doses of *P. histicola*). Clinical scores were assessed daily for the duration of the experiment. Flow cytometric plots of IL17^+^ or IFN*γ* ^+^-expressing mononuclear lymphoid cells were isolated from the brain and spinal cord of mice from all groups. Cells were isolated and stimulated with antigen (PLP_91–110_) plus Brefeldin A for 12 h. **(A)** Repregentative flow cytometric plots to demonstrate CD4^+^IL17^+^T cells, and CD4^+^IFN*γ* ^+^ T cells in the CNS of mice treated as above. **(B)** Quantification of the frequency of CD4^+^IL17^+^T cells, and CD4^+^IFN*γ* ^+^ T cells **(C)** from mice treated as in A. Cells were first gated on lymphocytes, singlets, and CD4^+^ cells. Gating strategy for different population had been provided in **Supplementary Figure 8**. The data presented are the average of two independent experiments with n ≥ 4 mice per group. The *p*-value determined by Mann-Whitney unpaired U test.

Next, we analyzed whether *P. histicola*, IFNβ, or the combination of both suppresses disease through influencing Th1 and Th17 cytokines. We isolated mononuclear cells from the brain and spinal cord of EAE mice from all groups and stimulated with the PLP_91–110_ peptide plus Brefeldin A for 14 h (28). HLA-DR3.DQ8 transgenic mice treated with *P. histicola* alone, IFNβ alone, the combination of *P. histicola* and IFNβ had a lower frequency of CD4^+^IL17^+^ T cells (**Figures 7A, B**) and CD4^+^IFN*γ* ^+^ T cells (**Figures 7C, D**) compared to those treated with media alone. Thus, our data suggest that treatment with *P. histicola* alone, IFNβ alone, the combination of *P. histicola* and IFNβ decreases the frequency of IFN*γ* ^+^ and IL17^+^ producing CD4^+^ T cells in the CNS of mice with EAE.

## Discussion

In the present study, we showed that *P. histicola* alone was as effective as the disease-modifying drug IFNβ in suppressing EAE in HLA-DR3.DQ8 transgenic mice. We also observed that the combination of *P. histicola* and IFNβ was not more effective compared to either treatment alone. The treatment with *P. histicola* alone or in combination IFNβ caused an increase in CD4^+^FoxP3^+^ Treg cells in the GALT of naïve HLA-DR3.DQ8 transgenic mice. Furthermore, we observed that *P. histicola* and/or IFNβ treatments effectively reduced microglia, astrocytes activation, and the frequency of IFN-γ and IL17- pro-inflammatory cytokine producing CD4^+^ T cells in the CNS of EAE mice. Thus, this study for the first time, showed that gut commensal *P. histicola* can suppress disease in HLA-DR3.DQ8 transgenic mice model of MS as effectively as IFNβ and both *P. histicola* and IFNβ utilize some common regulatory pathways including reduced activation of microglia, astrocytes and downregulation of pro-inflammatory immune response in the CNS.

The importance of *Prevotella* in MS can be highlighted by a number of studies showing a lower abundance of *Prevotella* in untreated MS patient, compared to healthy control (8–10, 14). Additionally, the abundance of *Prevotella* was increased in MS patients treated with disease-modifying drugs such as IFNβ and Copaxone (9, 15). We observed that a specific strain of *Prevotella* (*P. histicola*) can suppress disease in EAE mice. This is in line with our previous study, where we showed that *P. histicola* suppressed disease in a dose dependent manner (5). Our finding is further supported by the study showing that *P. histicola* can also suppress disease in an animal model of rheumatoid arthritis (29). Our study, for the first-time report that IFNβ can suppress disease in HLA-DR3.DQ8 transgenic mice. This is in agreement with earlier studies showing disease suppressive role of IFNβ in C57BL/6 (30) and SJL mice (31). Interestingly, we observed that a combination of *P. histicola* and IFNβ was not more effective in suppressing disease than either treatment alone. Previously, using Copaxone, we also did not observe any additive effect of *P. histicola* and Copaxone (6). One potential explanation for this phenomenon is that *P. histicola*, and IFNβ have the same or a similar mechanism of action, thus their effect may be convergent rather than synergistic.

CNS pathology is the hallmark of EAE/MS. Reduced inflammation and demyelination in *P. histicola* alone, IFNβ alone, and combination group (*P. histicola* plus IFNβ) indicate that disease suppression was accompanied with reduced CNS pathology. Lower disease in treatment groups was accompanied by lower microglia and astrocytes activation in the CNS. Enhanced activation of CNS resident microglia has been shown to play an important role in pathogenesis of EAE by promoting autoreactive T-cell responses through its ability to function as antigen presenting cells and neutralization of microglial activation can suppressed the development of EAE (25, 32–34). Similarly, reactive astrocytes are the main source of pro-inflammatory cytokine that plays a critical role in breaching the blood brain barrier (BBB) and induce recruitment of pathogenic immune cells into the CNS during MS and EAE (35–37). Astrocytes depletion is associated with reduced inflammation and demyelination in EAE mice (38). IFNβ and/or *P. histicola* can modulate neuroinflammatory properties of microglia and astrocytes by reducing Th17 cells infiltration into the CNS, as a higher CNS infiltration of Th17 cells can augment their neuroinflammatory properties of microglia and astrocytes (26). Thus, our data suggest that IFNβ and/or *P. histicola* induced anti-inflammatory environment might reduce microglia and astrocytes activation resulting in disease suppression.

MS and EAE are mediated by pro-inflammatory Th1 and Th17 T-cells (39–43). Reduced level of IL17^+^, and IFNγ^+^ CD4 T cell in the CNS of EAE mice are in line with current hypothesis that Th1 and Th17 play a pathogenic role in EAE (40, 44). Previously we have shown that *P*. *histicola* alone or combination with disease modifying drug Copaxone decreased IL17^+^ and IFN*γ^+^* CD4^+^ T cells infiltrating the CNS of HLA-DR3.DQ8 mice (6). Further, IFNβ treatment had been shown to reduce Th1 and Th17 cells as well as other inflammatory cytokines (45). Additionally, IFNβ treatment can inhibits IL-17 differentiation and induces IL-10 secretion in the T cells from the MS patients (46). Thus, our study suggests that *P. histicola* and IFNβ both suppressed disease by reducing pathogenic Th1 and Th17 cells in the CNS of EAE mice.

A number of therapeutic interventions in EAE had been shown to work through induction of Tregs and IL-10. We observed that *P. histicola* alone or in combination with IFNβ induced CD4^+^FoxP3^+^ Treg cells in the GALT of mice. Previous studies have shown that a single bacterium *P. histicola*, or *B. fragilis*, or a mixture *of Lactobacillus* species, or a mixture of *Clostridium* species can suppress EAE disease by inducing CD4^+^FoxP3^+^ Treg cells (5, 6, 47–49). Interestingly, we found that IFNβ alone treatment does not affect Treg cells in the gut. While IFNβ is associated with an increase in anti-inflammatory cytokines and reduced trafficking across the BBB, IFNβ is not associated with a modulation of Treg populations in mice (50). This is in contrast with effect of IFNβ in RRMS patients, where IFNβ treatment caused an increase in PD1^−^ Treg cells compared with PD1^+^ Treg in peripheral blood and CSF (51). The difference in our findings and Saresella et al. may be due to physiological differences between human and mice. Altogether, our study suggests that *P. histicola* can mediate disease suppressive effect through the induction of CD4^+^FoxP3^+^ Treg cells. Although the mechanism through which *P. histicola* induce Treg is not well understood. We hypothesize that *P. histicola* can induce Tregs in the gut through metabolism of dietary compounds (52).

In summary, our study suggests that *P. histicola* and IFNβ possesses both overlapping as well as non-overlapping modes of action in HLA-DR3.DQ8 transgenic mice. For example, *P. histicola* or IFNβ or combined treatment reduced the level of pro-inflammatory Th1 and Th17 cells, and reduced microglia and astrocytes activation in CNS of EAE mice but only groups receiving *P. histicola* induced anti-inflammatory Treg cells in the GALT. In conclusion, our present study, for the first time report that human gut commensal bacteria *P. histicola* suppresses EAE in HLA-DR3.DQ8 transgenic mice as effectively as commonly used MS drug IFNβ. As the gut microbiota seems to play an important role in the pathobiology of MS, beneficial gut bacteria such as *P. histicola* can provide additional treatment option for MS as well as other autoimmune inflammatory diseases.

## Data Availability Statement

The original contributions presented in the study are included in the article/**Supplementary Material**. Further inquiries can be directed to the corresponding author.

## Ethics Statement

The animal study was reviewed and approved by the Institutional Animal Care and Use Committee at the University of Iowa. Written informed consent was obtained from the owners for the participation of their animals in this study.

## Author Contributions

AKM conceptualized the study, designed and performed the experiments, and gave final approval of the manuscript to be published. SKS designed and performed the experiments, analyzed the data, and wrote the manuscript; SNJ helped with experimental design and performing experiment. ACM performed mouse genotyping, KG-C performed all histopathology and immunostaining. NT and HG performed western blot experiments. JZ, JM, and NK helped with the study design and interpretation of the data. All authors contributed to the article and approved the submitted version.

## Funding

The authors acknowledge funding from the National Multiple Sclerosis Society (RG 5138A1/1T), NIAID/NIH (1R01AI137075-01), a Carver Trust Medical Research Initiative Grant, and the University of Iowa Environmental Health Sciences Research Center, NIEHS/NIH (P30 ES005605). SF was supported on an institutional training grant (T32AI007485 to Dr. Gail Bishop) and diversity supplement award to AKM on patent 1R01AI137075.

## Conflict of Interest

AKM and JM are inventors of a technology claiming the use of *Prevotella histicola* for the treatment of autoimmune diseases. The patent for the technology is owned by Mayo Clinic, who has given exclusive license to Evelo Biosciences. AKM and JM received royalties from Mayo Clinic (paid by Evelo Biosciences).

The remaining authors declare that the research was conducted in the absence of any commercial or financial relationships that could be construed as a potential conflict of interest.
