# Supplementary material for: Human Commensal Prevotella histicola Ameliorates Disease as Effectively as Interferon-Beta in the Experimental Autoimmune Encephalomyelitis
Source: Front Immunol. 2020 Dec 11;11:578648. doi: 10.3389/fimmu.2020.578648 (PMC7759500; doi:10.3389/fimmu.2020.578648)
Supplement: Supplementary file 1 [file DataSheet_1.pdf]

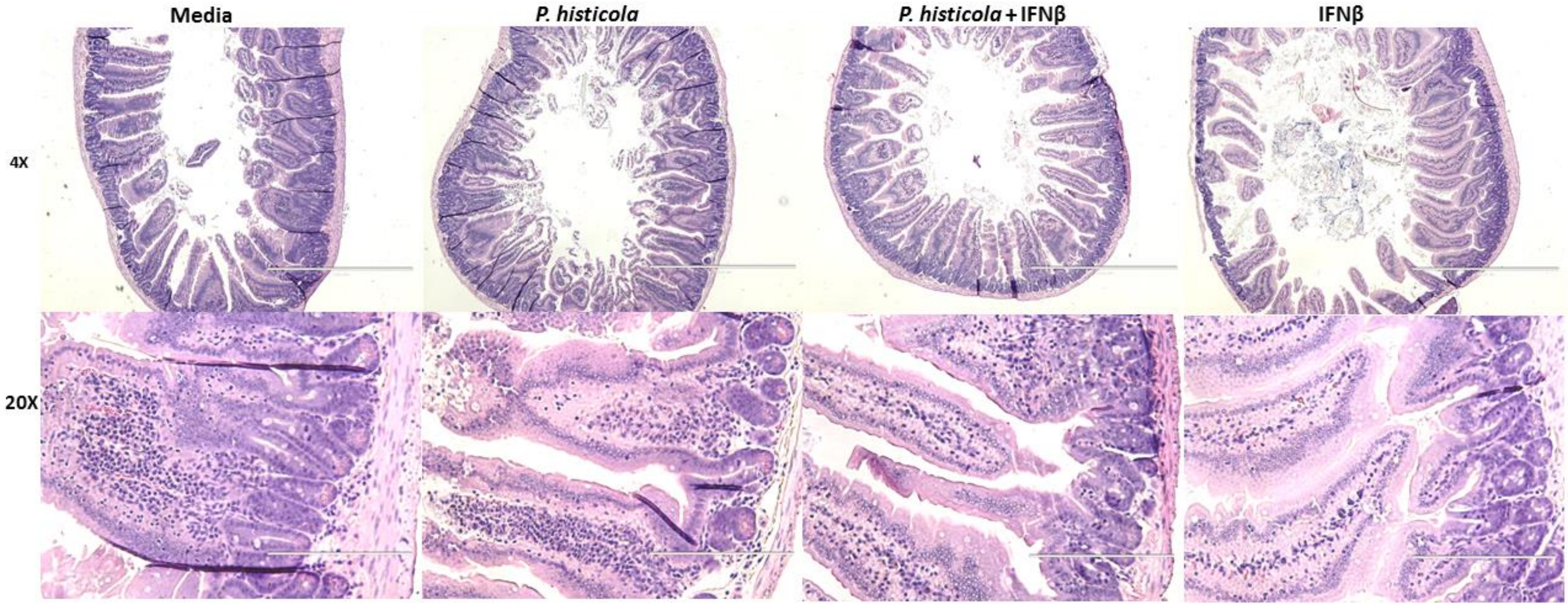


**Supplementary Figure 1: Treatment with *P. histicola* alone, IFNβ alone, and combination of *P.histicola* plus IFNβ did not caused pathology in the upper gut.** HLA-DR3.DQ8 transgenic mice were immunized with $PLP_{91-110}$ and were either treated with *P. histicola*, IFNβ, and combination of *P. histicola* plus IFNβ or medium staring at 7 days post-immunization for a total of 7 doses. Small intestinal tissue of Media, *P. histicola*, *P. histicola* and IFNβ combination and IFNβ treated mice were stained with hematoxylin and eosin.

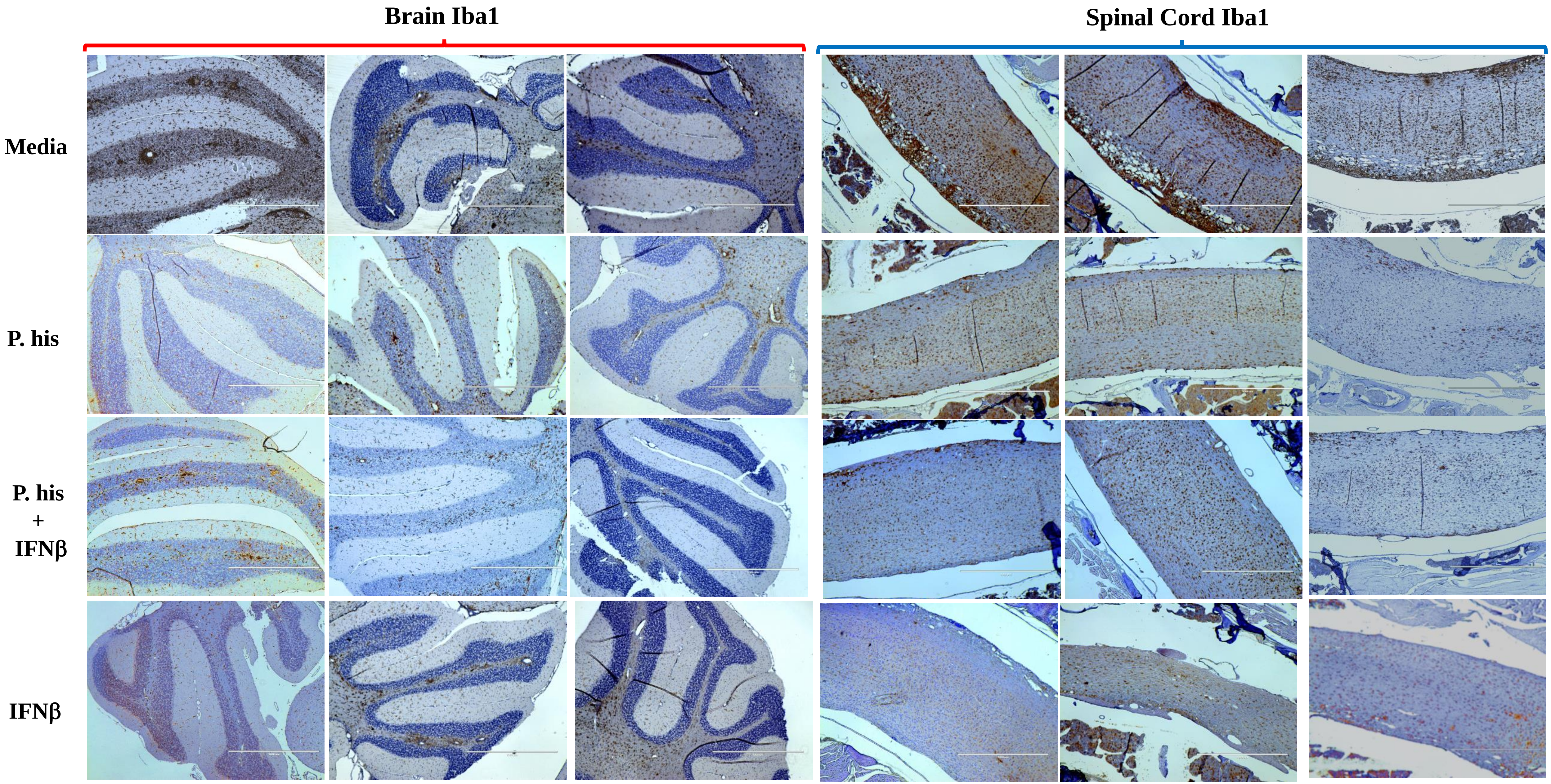


**Supplementary Figure 2. *P. histicola* alone, IFNβ alone, or the combination of *P. histicola* and IFNβ resulted in decreased microglial activation in the brain and spinal cord of mice with EAE.** Ionized calcium-binding adaptor protein-1 (Iba-1) staining of the brain (left three columns) and spinal cord sections (right three columns) from mice induced with EAE that received treatment with either *P. histicola* alone, IFNβ alone, the combination of both treatments, or media. The data presented at 4X resolution.

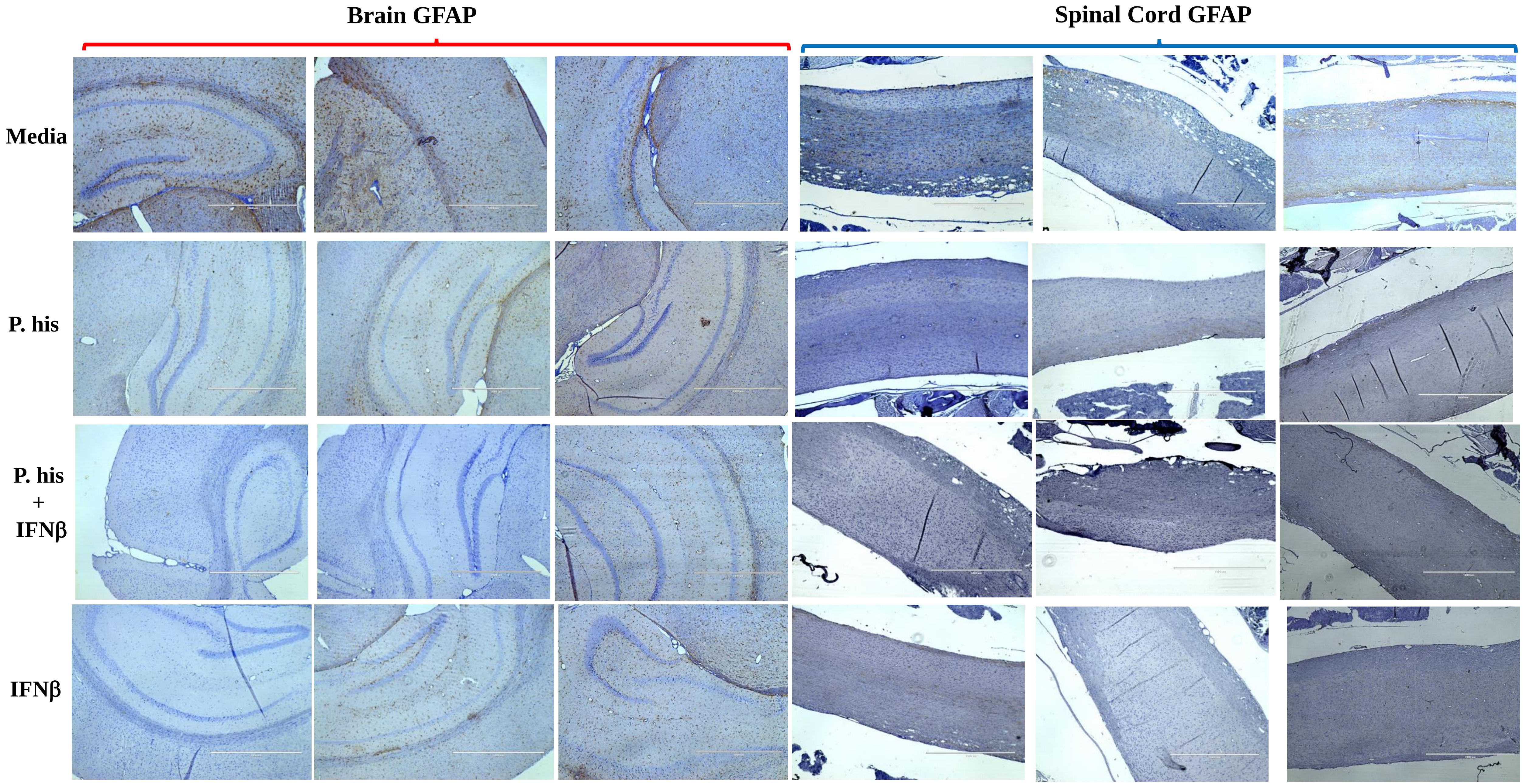


**Supplementary Figure 3.** ***P. histicola*** **alone, IFNβ alone, or the combination of** ***P. histicola*** **and IFNβ treatments reduced astrocytes activation in the brain and spinal cord of mice with EAE. A)** Staining glial fibrillary acidic protein (GFAP) of the brain (left three columns) and spinal cord sections (right three columns) from mice induced with EAE that received treatment with either *P. histicola* alone, IFNβ alone, the combination with IFNβ, or media only. The data presented at 4X resolution

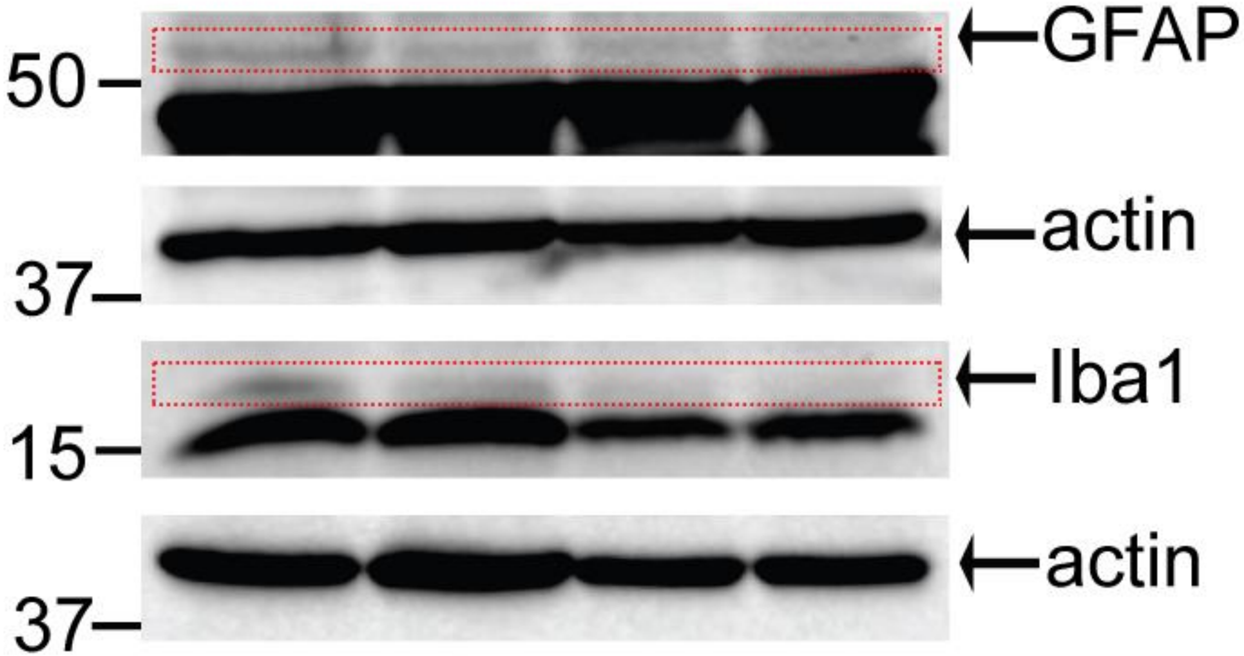


**Supplementary Figure 4. Treatment with *P. histicola* alone, IFNβ alone, or the combination of *P. histicola* and IFNβ reduced GFAP and Iba1 protein expression in spinal cord of mice with EAE.** The spinal cord's tissues from mice with EAE were collected and lysed in radioimmunoprecipitation assay (RIPA) buffer. Protein concentrations were determined using NanoDrop Spectrophotometer. 200 µg proteins were separated by SDSPAGE and transferred to nitrocellulose membranes expression of GFAP and Iba1 were analyzed using anti-GFAP and anti-Iba1 antibody respectively. Representative protein expression of GFAP and Iba-1of the spinal cord tissue from mice induced with EAE that received treatment with either *P. histicola* alone, IFNβ alone, the combination with IFNβ, or media only.

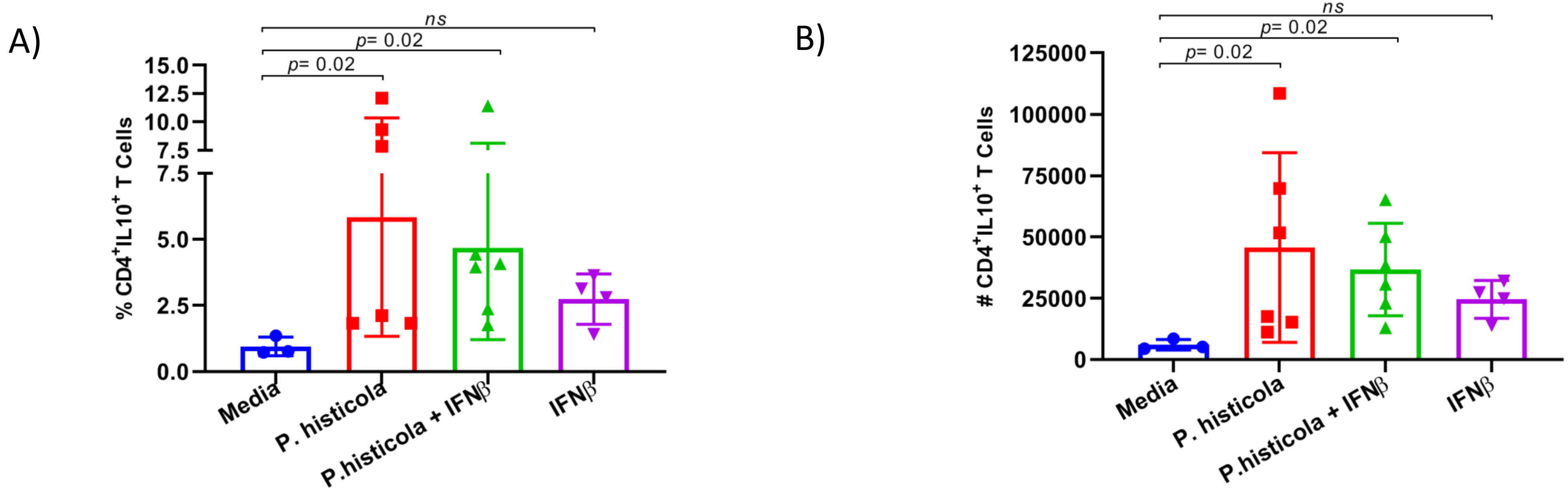


**Supplementary Figure 5. Pre-treatment with *P. histicola* alone or in combination with IFNβ increases CD4$^{+}$IL10$^{+}$ T cells in the gut-associated lymphoid tissue (GALT). A)** Naïve mice were treated with IFNβ (7 doses), *P. histicola* (7 doses), or a combination of both (with treatment administered on alternate days for a total of 14 doses). Gut-associated lymphoid cells were isolated from treated and the control group of mice and stained with CD45, CD4, and IL10 antibodies. Frequency of CD4$^{+}$IL10$^{+}$ T cells from mice treated as mentioned. **B)** Quantification of the number of CD4$^{+}$IL10$^{+}$ regulatory T cells in mice treated as in A. Error bars are presented as the standard error of the mean. The *p*-value determined by the Mann-Whitney unpaired U test for comparing each group to media.

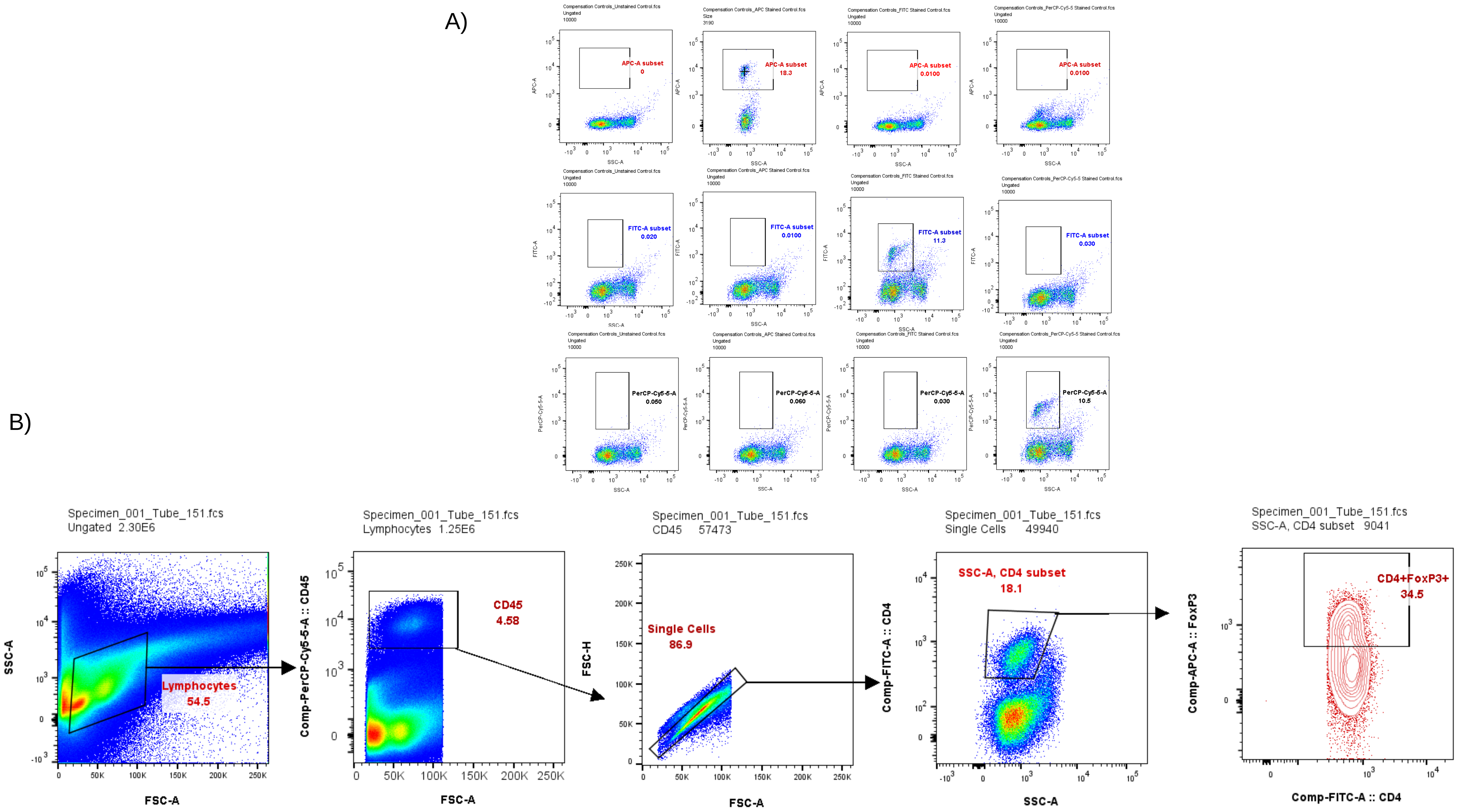


**Supplementary Figure 6:** Criteria for setting positive gates for specific fluorochrome FoxP3 (A) and a representative gating strategy for different cell populations isolated from gut-associated lymphoid tissue (B).

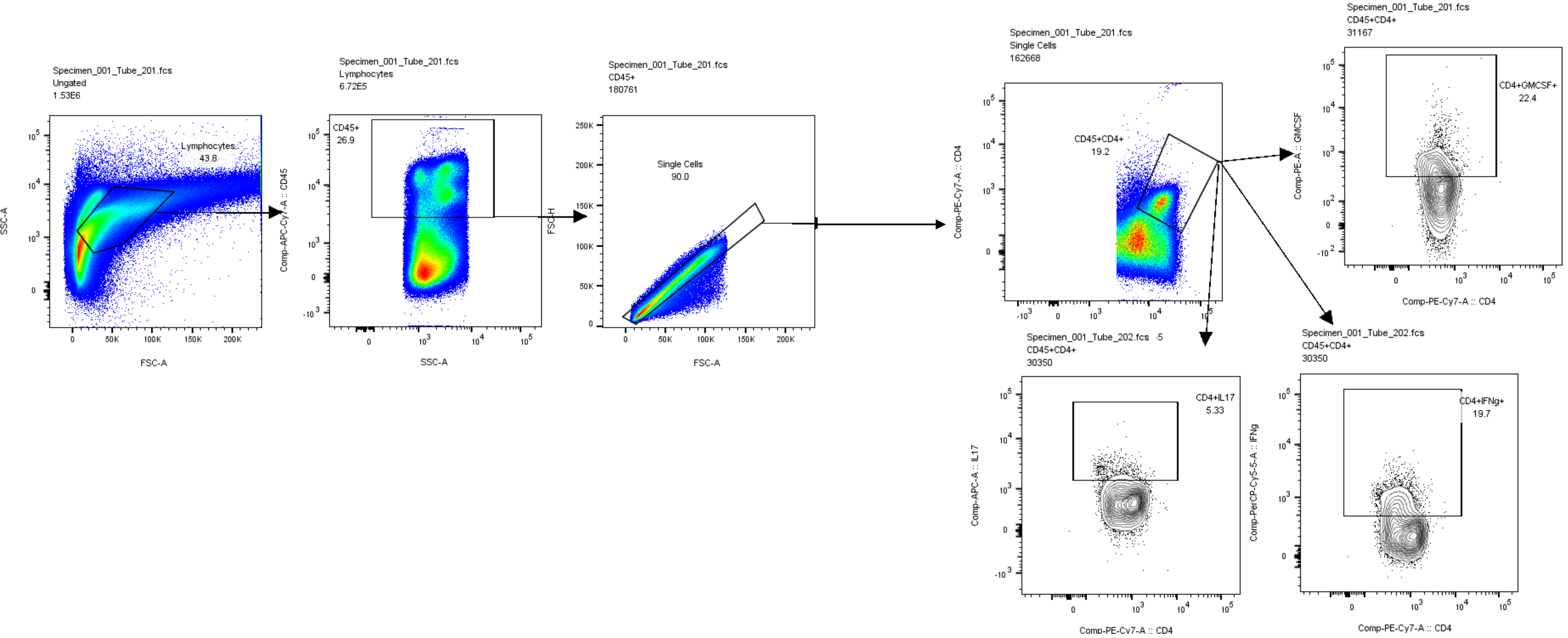


**Supplementary Figure 7**: Representative gating strategy for different population (IL17+, IFNγ+, and GM-CSF $^{+}$ CD4 T Cells) isolated from gut-associated lymphoid tissue.

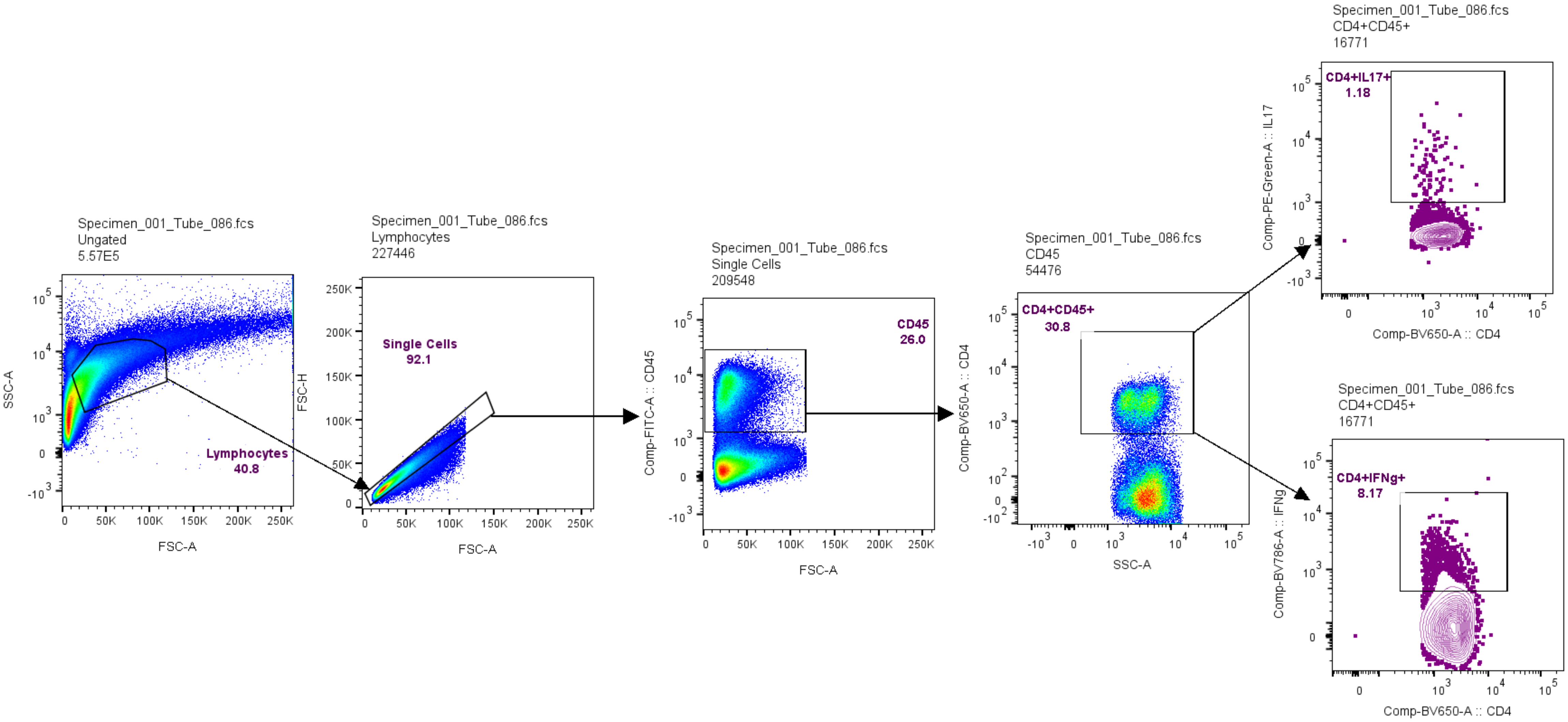


**Supplementary Figure 8:** Representative gating strategy for different population (IL17$^{+}$ and IFNγ$^{+}$ CD4 T Cells) isolated from CNS.
